# Fine mapping of the gene controlling the weeping trait of *Prunus persica* and its uses for MAS in progenies

**DOI:** 10.1186/s12870-022-03840-1

**Published:** 2022-09-24

**Authors:** Luwei Wang, Lei Pan, Liang Niu, Guochao Cui, Bin Wei, Wenfang Zeng, Zhiqiang Wang, Zhenhua Lu

**Affiliations:** National Peach and Grape Improvement Center/Key Laboratory of Fruit Breeding Technology of Ministry of Agriculture, Zhengzhou Fruit Research Institute, Chinese Academy of Agricultural Sciences, Zhengzhou, 450009 China

**Keywords:** Branch angle, Weeping, BSA-seq, Peach

## Abstract

**Background:**

Fruit tree yield and fruit quality are affected by the tree’s growth type, and branching angle is an important agronomic trait of fruit trees, which largely determines the crown structure. The weeping type of peach tree shows good ventilation and light transmission; therefore, it is commonly cultivated. However, there is no molecular marker closely linked with peach weeping traits for target gene screening and assisted breeding.

**Results:**

First, we confirmed that the peach weeping trait is a recessive trait controlled by a single gene by constructing segregating populations. Based on BSA-seq, we mapped the gene controlling this trait within 159 kb of physical distance on chromosome 3. We found a 35 bp deletion in the candidate area in standard type, which was not lacking in weeping type. For histological assessments, different types of branches were sliced and examined, showing fiber bundles in the secondary xylem of ordinary branches but not in weeping branches.

**Conclusions:**

This study established a molecular marker that is firmly linked to weeping trait. This marker can be used for the selection of parents in the breeding process and the early screening of hybrid offspring to shorten the breeding cycle. Moreover, we preliminary explored histological differences between growth types. These results lay the groundwork for a better understanding of the weeping growth habit of peach trees.

**Supplementary Information:**

The online version contains supplementary material available at 10.1186/s12870-022-03840-1.

## Background

Plant growth structure plays an important role in the economic value of crops, and it can indirectly affect yield and fruit quality. A tree structure is mainly determined by height, the growth direction of the branches, and the size and angle of the branches [1], which together determine the internal structure of the tree crown. With the continuous development of the fruit industry, efforts are made to develop novel variants and growth types that are widely adaptable, easy to manage and operate with mechanized means. Therefore, the molecular mechanisms regulating tree structure have attracted considerable research interest in the recent past. Plant structure is predominantly affected by environmental factors such as light, nutrients, and space size, however, genetic factors also play a major role [2–6]. Therefore, studying the genetic factors underlying tree structure may help improve fruit tree breeding and selection.

Due to the advantages of short growth cycles and simple handling, considerable progress has been made regarding research on the morphological development of herbaceous plants. Such studies have shown that mutations in the TAC1 (*Tiller angle control 1*) gene elicit vertical-like growth in plants such as rice (*Oryza sativa*) [7], and maize (*Zea mays*) [8]. Mutation of *LAZY1* (Regulator of nonsense transcript protein) causes Arabidopsis [9], rice [10], and maize [11] to produce horizontal lateral branches or tillers due to reduced lateral auxin transport. Three genes related to tiller angle and/or leaf angle were identified in cereal crops, i.e., Tiller angle control 1 (*TAC1*) and the two zinc finger proteins *LPA1* (Tetratricopeptide repeat-containing protein) and *PROG1* (PROSTRATE GROWTH 1) (Zinc finger protein GIS3) [7, 12]. *LPA1* and its Arabidopsis homolog *SGR5* (C2H2-like zinc finger protein) regulate geotropism upstream of amyloid deposition [13, 14]. However, the respective functional aspects underlying morphogenesis mechanisms in woody plants are less studied due to factors such as the plants’ large size, long growth cycles, and difficulty in establishing sufficiently large experimental colonies.

The weeping branch is one of the most important architectural plant traits, which is frequent in ornamental plants and is valued by breeders. Among Rosaceae, many tree species such as cherry blossoms, cherries, plums, begonias, and peach trees may occur as weeping branch types. Such trees are valued for landscaping and also important in scientific research on woody plant morphology [15]. Peach (*Prunus persica*) originates from China and is one of the largest groups of deciduous fruit trees. There are abundant peach genetic resources of different growth types in China, including standard type, pillar type, dwarf type, semi-dwarf type, and spur type. Weeping type peach trees were suggested to exhibit improved light transmission, which is conducive to fruit coloring and quality formation [16]. Peach trees show considerable annual growth, and the canopy is frequently closed during the fruit growth and development period, which is not conducive to ventilation and light transmission and may affect peel coloration. Weeping type trees present large opening angles, and limited pruning is sufficient to achieve a favorable and ornamental crown structure. In the past few years, many studies examined the genetic mechanism of different tree architectures. Dardick et al. (2013) used high-throughput sequencing to identify the candidate gene *Br* in peaches, which promotes horizontal branch growth. This gene is highly homologous to Tiller angle control 1 (*TAC1*) in rice and was thus named *PpeTAC1*. Molecular identification showed that the standard genotypes are *BrBr*, upright (*Brbr*), and pillar (*brbr*). Columnar (*br*) peach trees produce branches that grow nearly vertically, caused by a functional mutation of Tiller angle control 1 (*TAC1*). This gene encodes a protein belonging to the *IGT* gene family, which also includes *LAZY1* and *DRO1* (Deeper rooting 1) which regulate the orientation of lateral branches and roots, respectively [17]. RNAi silencing of Tiller angle control 1 (*TAC1*) in plums results in a branching orientation similar to that of columnar peach, however, with a narrower canopy. By contrast, overexpression of *PpeTAC1* in plum trees results in wider branching angles, more horizontal branching orientation, and formation of a larger crown [18]. Moreover, the gibberellin acid receptor gene (*PpeGID1c*) on the distal end of chromosome 6 is associated with peach dwarfing traits, and nonsense mutations in this gene lead to peach dwarfing [19]. A temperature-sensitive semi-dwarf mutant peach tree was observed to produce extremely short internodes at temperatures below 30 °C, and this mutant was used to establish an experimental population for locating the candidate gene that regulates the phenotype within the physical distance of 507.3 kb on scaffold 3 [20]. Moreover, genetic interactions between the columnar and weeping growth forms of peach trees were examined, showing that the columnar genotype (*PI*) had an epistatic effect, relative to the weeping genotype (*pl*), and an arching growth form (AR) appeared in the F_2_ generation of these two genotypes [21].

The genetic characteristics and regulatory mechanisms of weeping traits have been studied in crape myrtle (*Lagerstroemia indica* L.) [22], chestnut (*Castanea mollissima* Bl.) [23], and Japanese apricot (*Prunus mume*) [24], whereas little information on peach trees is available. In this study, we confirmed that the peach weeping branch growth type is a single-gene recessive trait. We applied a reduced-representation genome sequencing strategy to identify SNPs linked to the weeping locus derived from the peach accession ‘05–2-144’. Markers were developed for marker-assisted breeding, and a small chromosomal region was identified that contains 28 candidate genes. The similarities and differences among different types of shoots were analyzed at the cellular level using paraffin sections. The development of molecular markers for identifying weeping growth in peach trees helps provide important information for the efficient establishment of this trait in breeding, which will considerably improve the horticultural value of future peach varieties.

## Results

### Morphological and histological characterization

**Fig. 1:**
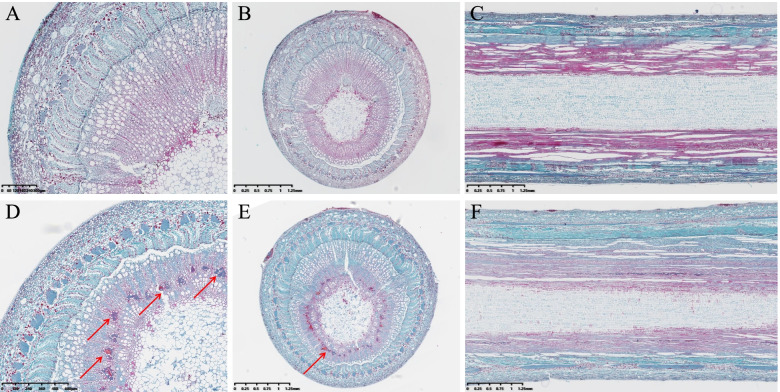
Paraffin sections of standard and weeping type branches. **A**, **B**: annual shoot cross-section of the weeping-type branch; (**C**): annual shoot longitudinal section of the weeping-type branch; (**D**, **E**): annual shoot cross-section of standard type; (**F**): annual shoot longitudinal section of weeping type

Cross and longitudinal sections of different growth types fixed in paraffin collected during the same period were compared (Fig. 1), showing that the organization was roughly similar, and the changing trend in different growth periods was the same. Compared with the weeping type, mature branches of the standard type showed obvious fiber bundles in the secondary xylem, and xylem fiber bundles were more abundant on the underside and less so on the upside.

### Trait mapping using BSA-seq analysis

The raw sequence data (Q20 ≥ 94.11%; Q30 ≥ 90.51%; GC content approximately 39.42–39.61%) of the standard and weeping pools amounted to 31.21 and 28.35 GB, respectively; after trimming the barcodes, the respective amounts of clean data were 31.14 and 28.28 GB. A total of 197,372,658 reads (104.24 folds average sequencing depth) and 174,956,782 reads (93.86 folds average sequencing depth) from the standard and weeping pools, respectively, were mapped to the reference genome (Table 1). Overall, the data were sufficient and showed good sequencing quality.

**Table 1:** Resequencing data of parental and pooled genomic samples

Sample	Mapped reads	Total reads	Mapping rate (%)	Average depth (-fold)	Coverage at least onefold (%)	Coverage at least fourfold (%)
yb144	174,956,782	193,088,680	90.61	93.86	99.07	98.69
ST	197,372,658	212,560,056	92.86	104.24	99.09	98.71
PL	174,956,782	193,088,680	90.61	93.86	99.07	98.69

yb144: 05–2-144 (Aa), Parent of the F_2_ generation; PL: weeping pool (aa); ST: standard pool (AA/Aa)

**Fig. 2:**
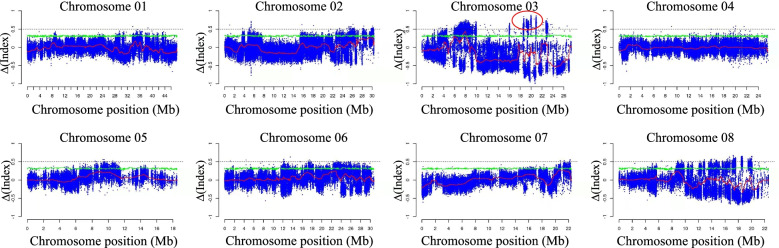
Candidate mapping region based on Delta SNP-index analysis. Green line: 95% confidence threshold; red line: Δ(SNP-index) distribution as a sliding window; the window above the confidence level is the candidate interval; blue coloration indicates the distribution of Δ(SNP-index) at each site

**Fig. 3:**
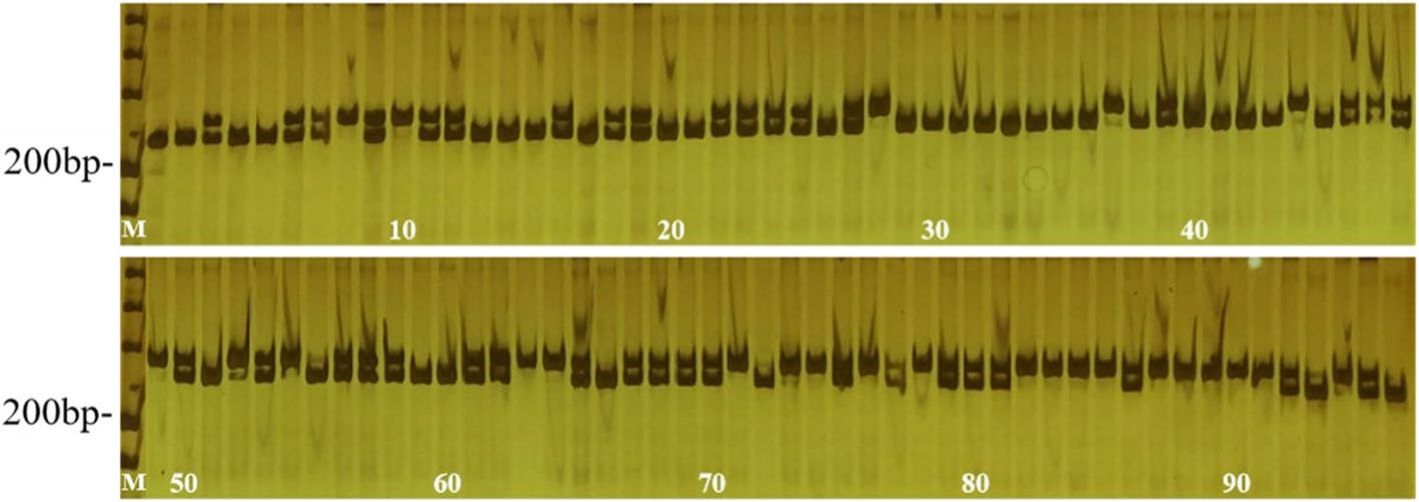
Validation of the gene locus controlling the weeping type based on BSA-seq analysis. M: DL Marker 500; The genotype of the single-band sample is homozygous, and the genotype of the two-band sample is heterozygous

Using parent ‘05–2-144’ as a reference, SNP indices (SNP frequency) of 641,993 marker sites between the two offspring types were examined. Trait mapping was performed by calculating the Δ(SNP-index) value between different progeny pools, selecting the F_2_ parent (genotype Aa) as a reference, and calculating the SNP-index (frequency of SNP) of the two gene pools respectively. SNP-index(weeping) = aa/Aa = 1,SNP-index(Standard) = AA/(AA + 2Aa) = 1/3,Δ(SNP-index) = SNP-index(weeping)-SNP-index(Standard) = 1–1/3 = 2/3 = 0.667. A total of 1,000 permutation tests were performed, and a 95% confidence level was used as a screening threshold. Through analyzing the SNP index of each pool, we calculated the Δ(SNP-index) of the entire genome from chromosomes 1–8 (Tables S1, S2 and S3). The position of genes that associated with the target trait was mapped between 19 and 21 Mb on chromosome 3 (Fig. 2). An indel marker, tightly associated with the weeping trait, was identified at 3 Mb through BSA-seq analysis, and it was validated in 96 individuals of the F_2_ progenies of ‘05–2-144’ (Fig. 3).

### Fine-mapping of the locus

**Fig. 4:**
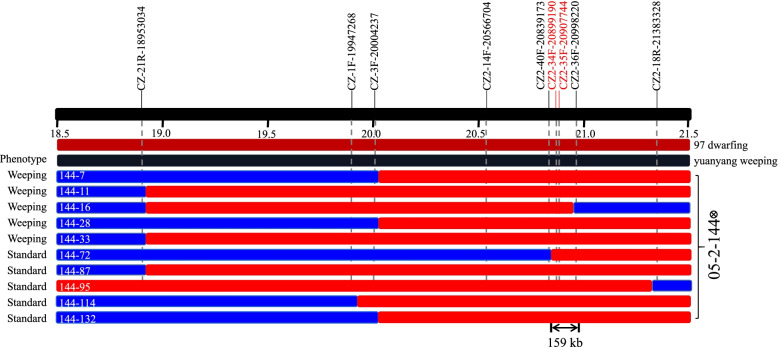
Fine mapping results of target genes controlling weeping traits in peach based on F_2_ segregating population. Red indicates the genotype-phenotype-linked region, and blue indicates that the two are not linked

Based on BSA-seq analysis, SNP and indel markers within the 3 Mb mapping interval on chromosome 3 were used to narrow down the mapping region (Table 2). First, primers were designed by selecting a variant site at a physical distance of approximately 0.5 Mb from the target interval (CZ-21R-18953034, CZ-1F-19947268, CZ2-SNP-14F-20566704, and CZ2-18R-21383328) to screen and count the number of recombinant individuals. The target gene was located on the right side of CZ-1F-19947268. To further identify candidate genes, primers for separation and screening were developed using IGV, according to the bam file. Nine pairs showed polymorphisms in parents and progeny, of which the two markers CZ2-34F-20899190 and CZ2-35F-20907744 were strongly linked to the trait of interest. After genotyping, the recombinant was identified, and the gene was located in the 159 kb interval between CZ2-40F-20839173 and CZ2-36F-20998220, and 28 genes were located between *Pp3G198900* and *Pp3G201500* (Fig. 4) (Table S4).

**Table 2:** Primers used for mapping

Primer name	Sequence (5`–3`)		Site
CZ-21R-18953034	AAAGTTCTTGTGGTCCCAAAGG	CTTGGGTCTGGTTAACTCTCCT	A/G
CZ-1F-19947268	TGCCTACCGAATCCCAGCAG	ACAAAACTGACAGCACTCGGT	A/G
CZ-3F-20004237	AGTCCGAGTCGAACCAAGC	AGCAAGAACGCGAGACACC	C/G
CZ2-14F-20566704	TCTGCTACCTCCAATGACGGT	AGCATCCTCTCGAGAACTTTCCT	T/C
CZ2-40F-20839173	GTGTGTCTTCCGTCCGATATAGT	TCAAGATGCAACAGAACTAAAACA	C/G
CZ2-34F-20899190	TGAGATTGTTGTTTTAGGGCCA	GGAAACAATAGACCACAGCAACA	A/G
CZ2-35F-20907744	TTGATCTGTCTTTCAATGGTCCG	CCAGTTATTTCGTTACCACCCAA	C/T
CZ2-36F-20998220	TATAATCCCCTCAACCGCCATAA	TAACTGTTCCTTTCCTCTTCCCA	C/T
CZ2-18R-21383328	CCCCACCATCCATCCACTCT	GAATGCTGGCGATAGGGGTG	T/C

### Candidate gene analysis and development of fully linked markers

**Fig. 5:**
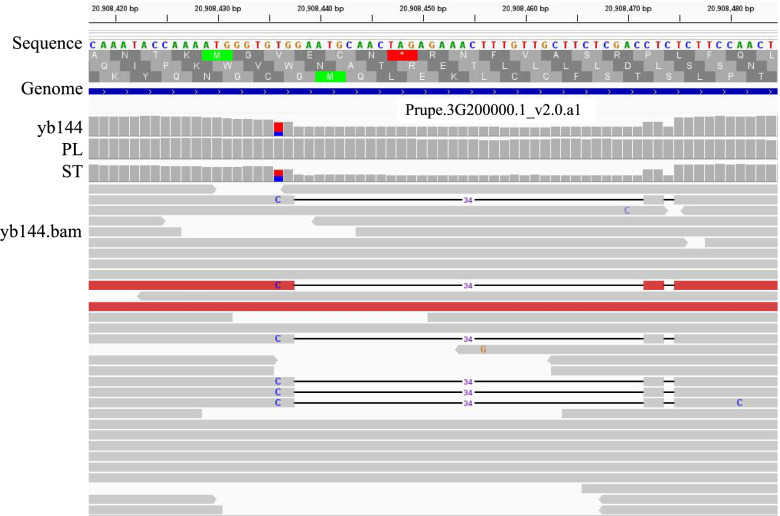
Alignment of bam files of the two growth types in IGV. yb144: 05–2-144 (Aa), F_2_ parent; PL: weeping pool (aa); ST: standard pool (AA/Aa)

**Fig. 6:**
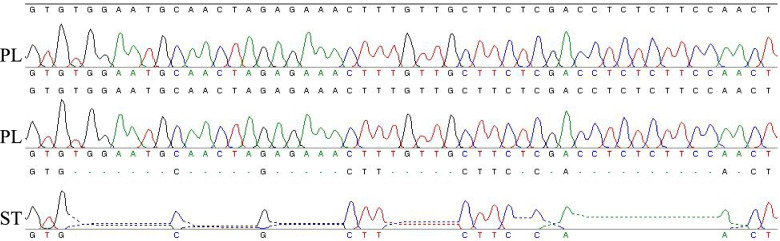
Accuracy of 35 bp deletions verified by Sanger sequencing. PL: weeping type (aa); ST: standard type (AA/Aa)

**Fig. 7:**
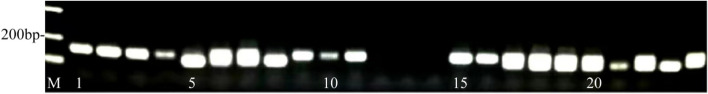
Validation results of 35 bp deletion in 24 recombinant individual plants. Note: 1–24 are recombinants; 1–4 and 9–16 are weeping types (aa); 5–8 and 17–24 are standard types (Aa or AA)

**Fig. 8:**
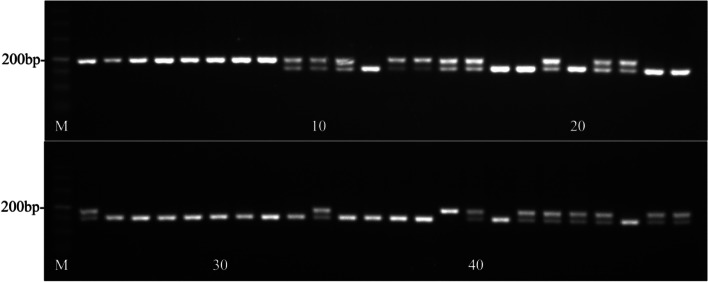
35 bp deletion validated in 48 commercial cultivars. Note: 1–8 and 39 are weeping types (aa); 5–8 and 17–24 are standard type (Aa or AA)

Using IGV to analyze the bam files of the standard type and the weeping type in the candidate region, a 35 bp heterozygous deletion was observed in the exon of *Pp3G20000* (Pp03-20,908,450) in the standard type (Fig. 5). Primers CZ-35deletion (F:5-CGAATTGCGCTTCTTACAACTT-3; R:5-CTGCCATGGAAGATTGATTGGA-3) were designed include the marker to examine the F_2_ generation population, the genotype and phenotype of individual plants were checked, and it was found that the genotypes and phenotypes of individual plants in the population were consistent, so it was determined that the marker was closely linked to the weeping trait (Figs. 6 and 7). The accuracy of the marker was further confirmed using 48 randomly selected commercial varieties, and the results show that this mark still applies (Fig. 8).

## Discussion

### Histology and endogenous hormones

Base and extension angles of tree branches are measured relative to the main stem, and the growth form of shoots, including extension direction and branching angle, is genetically determined, independent of gravity, and is further regulated by plant endogenous hormones [1, 25]. In a study on genetic control mechanisms in mutant plant development, it was shown that morphological development depends on co-regulation and interaction of endogenous hormones and related transcription factors [26].

A systematic analysis of the role of auxin in the formation of plant type suggested apical dominance in plant growth and development which is related to auxin anabolism, polar transport, and signal transduction [27]. Branch drooping may be caused in some plants by an altered geotropism response. A weeping mutant of Japanese morning glory shows the main stem morphology similar to that of the peach weeping phenotype [28, 29]; two alleles *weeping* (*we*1) and *weeping2* (*we2*) associated with stalk droop were found in morning glory mutants, which are highly homologous to *SCR* and *SHR* (GRAS family transcription factor) in Arabidopsis, respectively, and are found in different plants. The *GRAS* (A general term for a class of transcription factors in plants) family of transcription factors plays a crucial role in the development of the plant endothelium and the normal tropism response [30]. Cellulose in the cell wall acts as a scaffold for xylan and lignin during secondary wall formation and is critical for cell wall strength. In poplars with reduced cellulose synthase expression, the content of secondary cell wall cellulose was significantly reduced, while the content of lignin and non-cellulosic polysaccharides increased; the resulting weakened cell walls and insufficient support caused the branches of these trees to droop, which thus showed a similar to weeping peaches and cherries [31]. Plant secondary xylem provides support for shoot upward growth, and hormones regulate the differentiation of cambium cells in shoots to secondary xylem. Previous studies have shown that gibberellin is important for shoot elongation and phloem xylem development during which the level of GA_1_ (Gibberellin 1) correlates with the elongation of secondary xylem fibers [32]. A study on gibberellin synthesis in a gravity-responsive mutant of Japanese cherry blossoms suggested that gibberellin may affect the structure of the secondary xylem, thereby affecting the shoot’s gravity response [33].

Xiang et al.(2008) examined the levels of endogenous hormones in branches of different types of peach trees, and they found that the lignin content of straight branches was higher than that of drooping branches, and the lignin content of the underside and upside of the branches also differed between growth types. The data showed that the content of gibberellin on the upside of drooping peach branches was higher than that on the underside, which was consistent with the distribution of lignin. It was speculated that the uneven distribution of gibberellin may explain the unbalanced development of the secondary xylem [34]. Another study using transcriptional profiling of different organs of different growth types of crape myrtle showed that drooping shoots were deficient in endoderm cells, and GA_3_ (Gibberellin 3) treatment could rescue the weeping shoot phenotype. Transcriptional analysis and co-expression network analysis of differentially expressed genes indicated that gibberellin synthesis and signal transduction pathways play important roles in weeping traits. Virus-induced gene silencing of *LfiGRAS1*, a negative element of plant gibberellin signaling, leads to new branches growing in a negative direction, and gibberellin, interacting with other factors, strongly affects the growth type of crape myrtle [22].

In the present study, the cell morphology of two peach growth types was compared and analyzed, showing that the internal structure of the branches was dynamic, and the internal morphology of branches differed between growth types. Considering previous studies, we suggest that such differences are related to endogenous hormones; lignocellulose is an important substance providing support for branches, and a lack of lignocellulose in drooping peach branches is the main reason for branch drooping.

### Genetic characteristics and gene mapping of weeping peach

Monet described the weeping growth of peach trees as early as 1988, and Bassi later also studied weeping peach trees [35]. It was assumed that the weeping trait was recessive and was controlled by a single gene [19]. An analysis of several morphological traits of peach trees showed that weeping peach trees are beneficial for fruit harvesting [36]; the same study used 56 weeping type plants and 210 standard type plants in the F_2_ hybrid population of weeping peach, and the segregation ratio conformed to the 1:3 segregation ratio (*χ*^2^ = 2.2) of single-gene recessive traits according to Mendelian inheritance law. These results are consistent with the conclusions drawn from the preliminary population segregation of the present study.

The peach weeping gene (*pl*) of peach was located between two markers MA039a (17,772,071 bp) and SSRLG3_16m46 (21,811,873 bp) on LG3, with a physical distance of approximately 4 Mb [37]. Hollender et al. (2018) mapped the peach weeping growth-type gene at a physical distance of 435 kb (15,545,036–15,979,629 bp) on Pp03. A total of 56 annotated genes were included in this range. Combined with transcriptome data and sequence variation linkage identification, *Ppa013325* (*Prupe.3G200700*) was determined as the single candidate gene. The expression site of the gene was examined, showing that the gene was mainly expressed in the peach phloem, encoding a protein containing a sterility alpha motif domain, and the protein contained a total of 129 amino acids [18].

In the current study, based on BSA-seq, the candidate genes of the peach weeping branch were found to be located within a physical distance of 159 kb (20,839,173–20,998,220 bp) on chromosome 3, which was consistent with previous results [18, 37], however, the interval was comparably smaller. Hollender et al. (2018) verified the function of the *Ppa013325* gene in Arabidopsis and plum, and their results largely clarified the role of the gene in promoting shoot drooping. It was confirmed by qPCR (Quantitative Real-time PCR) that the gene was mainly expressed in the phloem of shoots; however, considering previous results on weeping branch-related endogenous hormones and morphological examination as performed in the current study, the main factor causing drooping in peach branches is a difference in the development of the xylem. In conclusion, the regulatory mechanism of the peach weeping trait requires further systematic study.

### Molecular markers linked to the peach weeping trait

Traditional breeding methods rely on the phenotype, the development of which, however, may take considerable time, thus impeding the breeding efficiency. Molecular genomic means may thus help improve and accelerate breeding selection. Molecular markers are not affected by the external growth environment and can be detected during the entire growth and development period of plants. Therefore, molecular markers closely related to late-stage plant development and growth traits are very important for the early selection of seedlings, which can greatly improve the breeding efficiency and shorten breeding time [23, 38]. Hollender et al. (2018) developed a KASP marker (AKSPP849) to detect deletion variants based on the identification of candidate genes, showing that the marker was homozygous in all weeping individuals and was deleted or absent in all non-weeping heterozygous individuals [18].

In the present study, based on fine mapping of genes associated with the weeping trait, sequence variation of candidate genes was further compared and analyzed. Primers were designed for confirmation in different populations, and a molecular marker was identified that is strongly linked to the candidate gene. This molecular marker can be used for early seedling identification or later gene function verification in the identification of target seedlings, thus representing a foundation for the development of new weeping peach varieties.

## Conclusions

Using the hybrid progeny ‘05–2-144’ of ‘97 dwarfing’ and ‘yuanyang weeping’, a total of 395 F_2_ generation populations were obtained. The target gene was located between 18–21 Mb on chromosome 3 based on BSA-seq, and fine-mapped in the range of 159 kb with SNP markers, using the 395 F_2_ individuals of this population. A candidate Indel in the gene *Prupe.3G200000* was obtained based on the resequencing data of other cultivars (lines). The Indel marker was able to identify the phenotypes in 72 cultivars (lines). By genotyping analysis, the Indel was co-segregated with the weeping phenotype and used for identification of stander or weeping type with 100% accuracy.

## Materials and methods

### Plant material

**Fig. 9:**
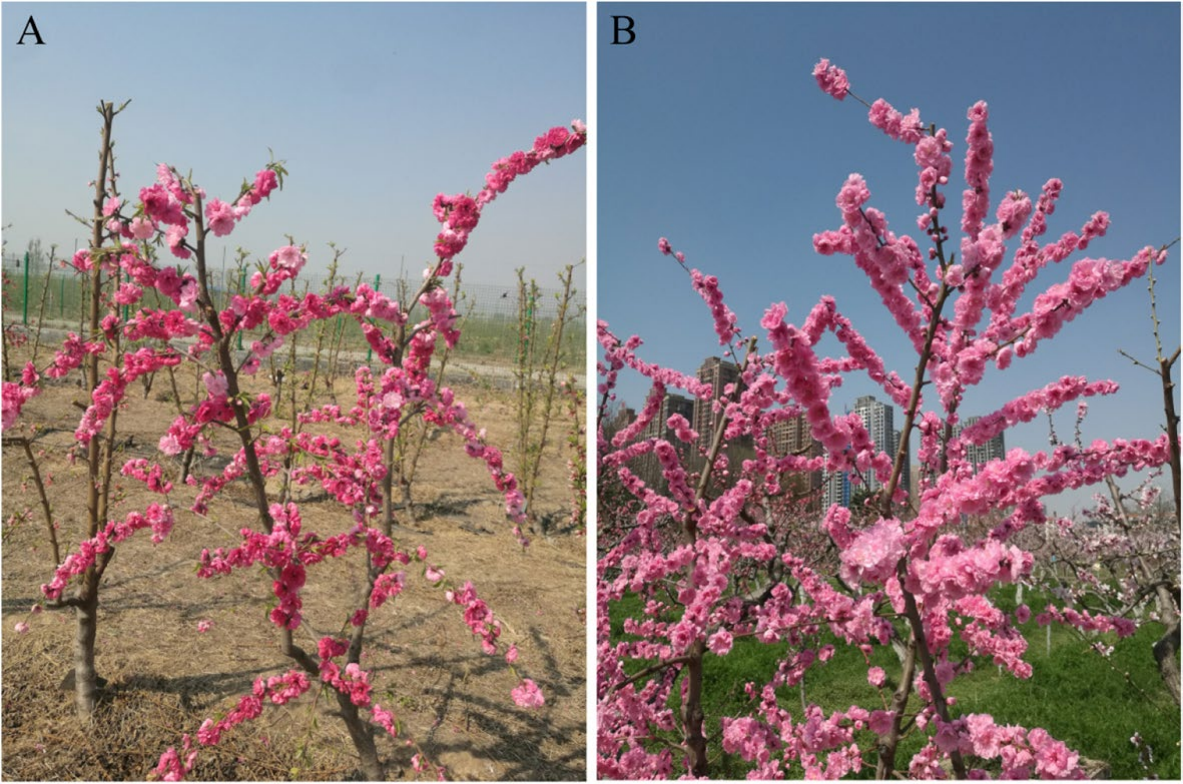
Growth types of plants of an F_2_ segregation population derived from peach accession ‘05–2-144’. **A** weeping type; (**B**) standard type

For gene mapping, the peach accession ‘05–2-144’ was used as a parent for inbreeding. Fruits were harvested in 2013, and peach pits were collected and stored in the sand after coating. In 2014, 395 seedlings of the F_2_ generation were obtained, which were planted in the peach breeding garden of Zhengzhou Fruit Tree Research Institute, Chinese Academy of Agricultural Sciences. Accession ‘05–2-144’ (Fig. 9) was derived from a selfed F_1_ individual that was the offspring of accessions ‘97 dwarfing’ and ‘yuanyang weeping’. The parent variety ‘97 dwarfing’ is a dwarf type, and ‘yuanyang weeping’ is a weeping type, resulting in standard-type offspring. In 2014 and 2015, F_2_ segregation populations were produced to acquire sufficient individuals for fine mapping.

### Morphological and histological characterization

After defoliation, the ‘crotch angle’ and the ‘extension angle’ were measured in one-year-old branches to distinguish the two different types of tree architecture. Five branches were measured per tree. For histological assessments, branches of each phenotype were sampled and were fixed in FAA solution (5% formaldehyde, 5% acetic acid, and 50% ethanol), samples were stored at 4 °C after vacuum drying.

The samples were dehydrated using the following treatments: (1) 75% ethanol for 4 h (2) 85% ethanol for 2 h (3) 90% ethanol for 2 h (4) 95% ethanol for 1 h (5) absolute ethanol for 1 h (6) transition into histo-clear xylene for 20 min (7) embedding in paraffin using TKY-BMB (JB-T5, Wuhan Junjie Equipment Factory, Wuhan, China). Thereafter, the samples were sliced (4 μm thickness) using a rotary microtome (Leica RM2016; Leica Microsystems, Germany). Three samples were examined per replicate.

### Genetic segregation analysis

In 2016, the phenotype of 395 offspring of the F_2_ population was determined by measuring the branching angle of each plant. The inbred progeny of ‘05–2-144’ had two types: standard type and weeping type. Statistical analysis of two single trees showed that the segregation ratio of the progeny population was close to 3:1 (standard type to weeping type), which was in line with Mendelian inheritance and with the expected ratio of the single-gene recessive control (*χ*^2^ = 0.19; *P* = 0.67), suggesting that the weeping phenotype was controlled by a single recessive gene.

### DNA extraction and quantification

Young leaves were collected and placed on the bottom of 1.2 mL eight-row centrifuge tubes using tweezers. Total genomic DNA was isolated using the CTAB method, with minor modifications [39]. Quantity and quality of total DNA were assessed using a Nanodrop spectrophotometer (Thermo Fisher Scientific, Waltham, MA, USA). Twenty DNA samples were mixed in equal ratios to generate standard and weeping-type genomic pools for high-throughput sequencing on an Illumina HiSeq platform (Illumina, San Diego, CA, USA) in paired-end mode (2 × 150 bp).

### Gene mapping based on BSA-seq

Based on the phenotypic evaluation, we selected 15 individual plants of the standard type and weeping type in 2017 to extract DNA. Fifteen samples of each phenotype were mixed in equal amounts, and SNP markers were developed through BSA-seq resequencing to preliminarily locate the target loci. Briefly, clean reads were first aligned to the peach reference genome (https://www.rosaceae.org/species/prunus_persica/genome_v2.0.a1) using a BWA aligner, then SAMTOOLS was used to convert alignment files to SAM/BWA files and to perform SNP calling [10]. SNP-calling was performed using GATK3.3 software [40].

SNP indices of both phenotypes were calculated for all positions as described previously to identify candidate regions associated with the weeping trait. Then, SNP indices were calculated for each DNA pool with 1 Mb intervals and a 1 kb sliding window. The parent ‘05–2-144’ was used for error elimination. The following criteria were used for SNP indexing: (1) sequencing depth below seven-fold and SNP index < 0.3 were discarded, and (2) Only one pool exists SNP missing were discarded. Therefore, a weeping candidate region had an SNP index of 1, and the standard SNP index = 1/3. The Δ(SNP-index) was obtained by subtracting the SNP index of the weeping pool from that of the standard pool, which was approximately 0.333.

### Marker validation and gene fine mapping based on resequencing

Based on BSA-seq, the candidate regions associated with the weeping trait were validated using the indel markers as confirmed through PAGE. For fine mapping, SNP markers were used for polymorphism screening in the parent of ‘05–2-144’ and the offspring. As for referencing the genome, IGV software was used to detect SNPs in the bam files. Primers were designed using Primer3 (available at http://primer3.ut.ee).

PCR amplification for Sanger sequencing was performed using a reaction volume of 40 μL, and with a 15 μL reaction volume for indel analysis. For indel markers, the PCR products were examined using PAGE and were Sanger sequenced for SNP analysis.

## Supplementary Information

**Additional file 1: Table S1.** Candidate region of SNP_index.

**Additional file 2: Table S2.** Genome-wide Delta_SNP_index.

**Additional file 3: Table S3.** Locus information of candidate gene.

**Additional file 4: Table S4.** Fine-mapping results based on segregated populations.

## Data Availability

The raw BSA-seq data were deposited in the NCBI data-base under accession number PRJNA726025 (https://www.ncbi.nlm.nih.gov/bioproject/726025).

## Abbreviations

BSA-seq: Bulked Sergeant Analysis Sequencing
KASP: Kompetitive Allele Specific PCR
PAGE: Polyacrylamide Gel Electrophoresis
PL: Weeping type
SNPs: Single-Nucleotide Polymorphisms
ST: Standard type

## References

[1] Tomlinson PB (1983). Tree architecture: new approaches help to define the elusive biological property of tree form. Am Sci.

[2] Halle F, Oldeman RA, Tomlinson PB (1978). Tropical trees and forest. An architectural analysis.

[3] Mehlenbacher SA, Scorza R (1986). Inheritance of growth habit in progenies of compact Redhaven peach. Hortic Sci.

[4] Scorza R, Bassi D, Liverani A (2002). Genetic interactions of pillar (columnar), compact, and dwarf peach tree genotypes. J Am Soc Hortic Sci.

[5] Barthelemy D, Caraglio Y (2007). Plant architecture: a dynamic, multilevel, and comprehensive approach to plant form, structure and ontogeny. Ann Bot.

[6] Busov VB, Brunner AM, Strauss SH (2008). Genes for control of plant stature and form. New Phytol.

[7] Yu B, Lin Z, Li H, Li X, Li J, Wang Y, Zhang X, Zhu Z, Zhai W, Wang X, Xie D, Sun C (2007). *TAC1*, a major quantitative trait locus controlling tiller angle in rice. Plant J.

[8] Ku L, Wei X, Zhang S, Zhang J, Guo S, Chen Y, Lustig AJ (2011). Cloning and characterization of a putative TAC1 ortholog associated with leaf angle in maize (*Zea mays L*.). Plos One.

[9] Yoshihara T, Iino M (2007). Identification of the gravitropism-related rice gene *LAZY1* and elucidation of *LAZY1*-dependent and -independent gravity signaling pathways. Plant Cell Physiol.

[10] Li H, Durbin R (2009). Fast and accurate short read alignment with burrows-wheeler transform. Bioinformatics.

[11] Dong Z, Jiang C, Chen X, Zhang T, Ding L, Song W, Luo H, Lai J, Chen H, Liu R, Zhang X, Jin W (2013). Maize *LAZY1* mediates shoot gravitropism and inflorescence development through regulating auxin transport, auxin signaling, and light response. Plant Physiol.

[12] Tan L, Li X, Liu F, Sun X, Li C, Zhu Z, Fu Y, Cai H, Wang X, Xie D, Sun C (2008). Control of a key transition from prostrate to erect growth in rice domestication. Nat Genet.

[13] Wu X, Tang D, Li M, Wang K, Cheng Z (2013). Loose plant architecture1, an indeterminate domain protein involved in shoot gravitropism, regulates plant architecture in rice. Plant Physiol.

[14] Tanimoto M, Tremblay R, Colasanti J (2008). Altered gravitropic response, amyloplast sedimentation and circumnutation in the Arabidopsis shoot gravitropism 5 mutant are associated with reduced starch levels. Plant Mol Biol.

[15] Ottesen C. The American Gardener. American; 2003. p. 46-50.

[16] Infante RP, Martínez Gómez, Predieri S (2008). Quality oriented fruit breeding: Peach [*Prunus persica* (L.) Batsch]. J Food Agric Env.

[17] Dardick C, Callahan A, Horn R, Ruiz KB, Zhebentyayeva T, Hollender CA, hitaker M, Abbott A, Scorza R (2013). *PpeTAC1* promotes the horizontal growth of branches in peach trees and is a member of a functionally conserved gene family found in diverse plants species. Plant.

[18] Hollender CA, Pascal T, Tabb A, Hadiarto T, Srinivasan C, Wang W, Liu Z, Scorza R, Dardick C (2018). Loss of a highly conserved sterile alpha motif domain gene (*WEEP*) results in pendulous branch growth in peach trees. P Natl Acad Sci USA.

[19] Hollender CA, Dardick C (2015). Molecular basis of angiosperm tree architecture. New Phytol.

[20] Lu Z, Niu L, Chagné D, Cui G, Pan L, Foster T, Zhang R, Zeng W, Wang Z (2016). Fine mapping of the temperature-sensitive semi-dwarf (*Tssd*) locus regulating the internode length in peach (*Prunus persica*). Mol Breeding.

[21] Werner DJ, Chaparro JX (2005). Genetic interactions of pillar and weeping peach genotypes. Hortic Sci.

[22] Li S, Zheng T, Zhuo X, Li Z, Li L, Li P, Qiu L, Pan H, Wang J, Cheng T, Zhang Q (2020). Transcriptome profiles reveal that gibberellin-related genes regulate weeping traits in crape myrtle. Horticult Res.

[23] Terakami S, Nishio S, Kato H, Takada N, Saito T, Yamamoto T (2021). Genetic mapping of the dominant gene controlling weeping habit in Japanese chestnut (*Castanea crenata* Sieb. et Zucc.). Tree Genet Genomes.

[24] Socquet-Juglard D, Christen D, Devènes G, Gessler C, Duffy B, Patocchi A (2012). Mapping architectural, phenological, and fruit quality QTLs in apricot. Plant Mol Biol Rep.

[25] Fisher JB, Honda H (1979). Branch geometry and effective leaf area: a study of Terminalia-branching pattern. II. Survey of real trees. Am J Bot.

[26] Pengtao G, Di L (2005). Genetic control of plant shoot branching. Mol Plant Breed.

[27] Wang B, Li J, Wang Y (2006). Advances in understanding the roles of auxin involved in modulating plant architecture. Bull Bot.

[28] Kitazawa D, Hatakeda Y, Kamada M, Fujii N, Miyazawa Y, Hoshino A, Iida S, Fukaki H, Morita MT, Tasaka M (2005). Shoot circumnutation and winding movements require gravisensing cells. Proc Natl Acad Sci USA.

[29] Kitazawa D, Miyazawa Y, Fujii N, Hoshino A, Iida S, Nitasaka E, Takahashi H (2008). The gravity-regulated growth of axillary buds is mediated by a mechanism different from decapitation-induced release. Plant Cell Physiol.

[30] Tasaka M, Kato T, Fukaki H (1999). The endodermis and shoot gravitropism. Trends Plant Sci.

[31] Joshi CP, Thammannagowdaa S, Fujino T, Gou J-Q, Avci U, Haigler CH, McDonnelle LM, Mansfielde SD, Mengeshaf B, Carpitaf N, Harrisg D, DeBolt S, Peter GF (2011). Perturbation of wood cellulose synthesis causes pleiotropic effects in transgenic aspen. Mol Plant.

[32] Ridoutt BG, Pharis RP, Sands R (1996). Fibre length and gibberellins A1 and A20 are decreased in Eucalyptus globules by acylcyclohexanedione injected into the stem. Physiol Plantarum.

[33] Teruko N, Maki S, Yukiko I, Reiko I, Sachiko H, Mayumi H, Yukio I (1994). The effects of GA3 on weeping of growing shoots of the Japanese Cherry. Prunus spachiana Plant Cell Physiol.

[34] Xiang S, Li Y, Kang L, Zou Y, Shu H (2008). Relationship between morphology and hormones during weeping peach. Acta Horticulturae Sinica.

[35] Monet R, Bastard Y, Gibault B (1988). Etude génétique du caractère port pleureur chez le pêcher. Agronomie.

[36] Dirlewanger E, Bodo C (1994). Molecular genetic mapping of peach. Euphytica.

[37] Pascal T, Aberlenc, Confolent C, Hoerter M, Lecerf E, Tuéro C, Lambert P. Mapping of new resistance (*Vr2*, *Rm1*) and ornamental (*Di2*, *pl*) Mendelian trait loci in peach. Euphytica. 2017;213(6):132.

[38] Chaparro JX, Werner DJ, O'Malley D, Sederoff R (1994). Targeted mapping and linkage analysis of morphological isozyme, and RAPD markers in peach. Theor Appl Genet.

[39] Doyle J, Doyle JL (1987). A rapid DNA isolation procedure for small quantities of fresh leaf tissue. Phytochem Bull.

[40] McKenna A, Hanna M, Banks E, Sivachenko A, Cibulskis K, Kernytsky A, Garimella K, Altshuler D, Gabriel S, Daly M, DePristo MA (2010). The genome analysis toolkit: a Map Reduce framework for analyzing next-generation DNA sequencing data. Genome Res.

